# Resistance to Hydrocyanic Acid Poisoning

**Published:** 1890-07

**Authors:** 


### RESISTANCE TO HYDROCYANIC ACID POISONING.

Dr. A. Jasme, of Savannah, Ga., reports a case of a horse eight years old, weighing about 1050 pounds, which he undertook to destroy on account of a fracture of the tibia. He injected 150 minims hypodermically, and in ten minutes fifty minims more ; no symptoms were then visible. Injections of twenty-five minims each were then repeated, until at the end of thirty-five minutes the horse had received a whole ounce of the poison carefully injected in the subcutaneous tissues of the neck and chest. No symptoms were visible until after the injection of 250 minims when the heart's action could be heard, standing at a distance from the horse. The pulse was full and increased to 70 ; the respirations were not disturbed ; there was but little excitement of the nervous system.

Forty minutes after the last injection the animal seemed in a perfectly normal condition, and had to be destroyed by other means. The acid used was dilute hydrocyanic acid, freshly prepared, and contained in a glass stoppered bottle, so that there was no question as to the quality of the drug.

